# Congenital Ulnar Drift in a Surgeon

**DOI:** 10.1155/2015/135350

**Published:** 2015-06-18

**Authors:** Desirae McKee, Shannon Eliasson, John Griswold

**Affiliations:** Department of Orthopaedic Surgery, Texas Tech University, 3601 4th Street, Mail Stop 9436, Lubbock, TX 79430-9436, USA

## Abstract

Windblown hand is a term used in many instances to describe ulnar deviations of the fingers with or without other malformations. In 1994 Wood reviewed all of the descriptions of cases of windblown hand and pointed out how many variants of congenital ulnar drift there are, suggesting that the many variations seen may all belong to a larger type of arthrogryposis. While the most common cause of ulnar deviation of the fingers is rheumatoid arthritis, it can also be caused by other conditions such as windblown hand or Jaccoud's arthropathy. While most hand surgeons are familiar with presentations of congenital ulnar drift, few of them are knowledgeable about Jaccoud's arthropathy as this is usually discussed within medical communities such as Rheumatology. We present a case of a surgeon who has had noticeable ulnar deviation of the digits at the level of the metacarpophalangeal joint since his early 20s. We propose that the current case is a demonstration of a type of windblown hand that has some hereditary component but is not immediately obvious at birth and presents physically more like Jaccoud's arthropathy than traditional windblown hand.

## 1. Introduction

Windblown hand is a term used in many instances to describe ulnar deviations of the fingers with or without other malformations. In 1994, Wood [1] reviewed all of the descriptions of cases of windblown hand and pointed out how many variants of congenital ulnar drift there are, suggesting that the many variations seen may all belong to a larger type of arthrogryposis. While the most common cause of ulnar deviation of the fingers is rheumatoid arthritis, it can also be caused by other conditions such as windblown hand or Jaccoud's arthropathy. While most hand surgeons are familiar with presentations of congenital ulnar drift, few of them are knowledgeable about Jaccoud's arthropathy as this is usually discussed within medical communities such as Rheumatology.

We present a case of a surgeon who has had noticeable ulnar deviation of the digits at the level of the metacarpophalangeal joint since his early 20s. Two brothers in the same family also had a history of ulnar deviation of the fingers that was not noticed until they were adults. At over 50 years of age, all of them have noticed the condition for nearly 30 years and none of them have ever received treatment for it. We propose that the current case is a demonstration of a type of windblown hand that has some hereditary component but is not immediately obvious at birth and presents physically more like Jaccoud's arthropathy than traditional windblown hand.

## 2. Case Report

**Figure 1:**
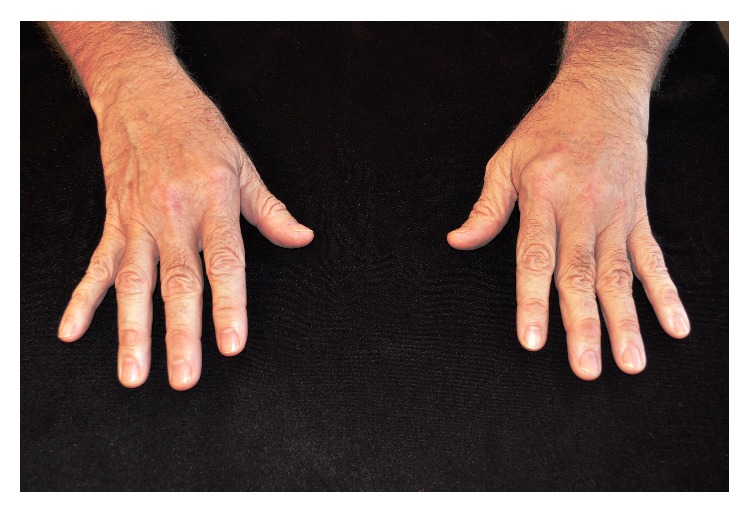
Clinical Photo of bilateral windblown hand.

**Figure 2:**
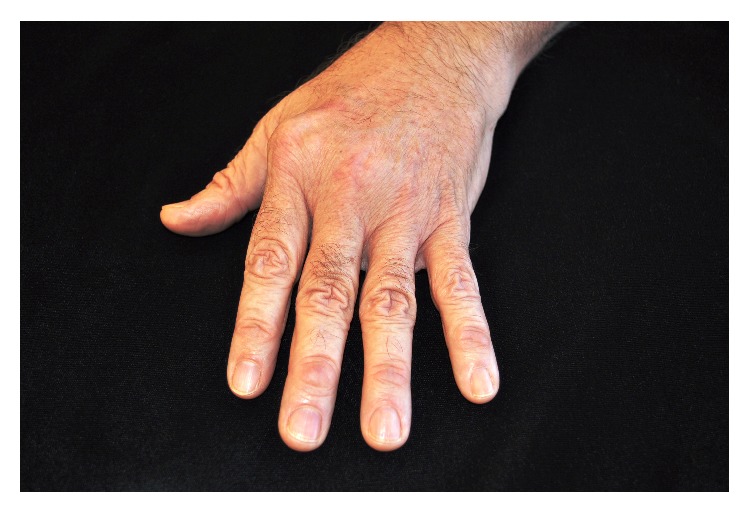
Clinical Photo of left windblown hand demonstrating ulnar drift of the digits at the MCP joint.

A 55-year-old Caucasian male presented with bilateral ulnar deviation of the fingers that could be corrected without pain (Figures 1 and 2). He first noticed the ulnar deviation in his early twenties. Initially he had no associated pain and he still had full function of his hands and therefore did not receive treatment. Three years prior to presentation he developed pain at the base of both thumbs and approximately 4 months priorly he began to have a significant increase in pain in his left 2nd and 3rd metacarpophalangeal (MCP) joints as well as in both 1st carpometacarpal (CMC) joints. He also began to notice some wasting in the muscles between his thumb and index fingers. As an active surgeon, he noted minor difficulty with some instrumentation such as hemostat use when in the OR. Symptomology was treated with over the counter anti-inflammatory medication.

The patient has two brothers (aged 54 and 53) that are affected, one brother that is not affected (age 45), and two sisters that are not affected. Both parents are deceased but the patient did not believe that they had any ulnar deviation of their fingers. The patient also has three children, none of which show hand abnormalities. By report, neither of the patient's affected brothers have any pain or other arthritic symptoms associated with their hands.

On physical exam bilateral adductor wasting was noted bilaterally with greater changes on the left hand. No symptoms of numbness in the ulnar nerve distribution were encountered. He has hyperextension of the PIP joints that is worse on the left side giving a mild swan neck impression of the digits. Light touch neurosensory was intact. He had a minor prominence at the right volar wrist that was clinically compatible with ganglion cyst formation secondary to CMC arthritis. CMC grinding was present bilaterally at the first metacarpal as well as bilateral enlargement of the first and second MCP with positive grind at the left 2nd MCP. He had a negative Wartenberg test and no interosseous wasting, and full extension was possible. Grip strength was R78 lbs and L62 lbs tested with dynamometer, while his pinch strength was R11 lbs and L8 lbs, respectively. No bands were palpable on the palm with extension and radial displacement of the fingers. No subluxation of the extensor tendon was noted.

**Figure 3:**
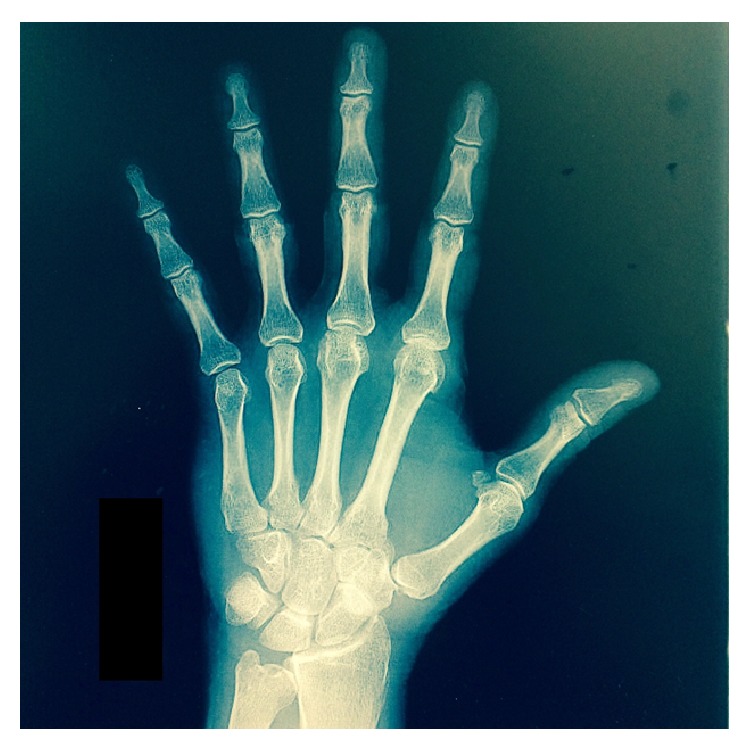
Left windblown hand.

**Figure 4:**
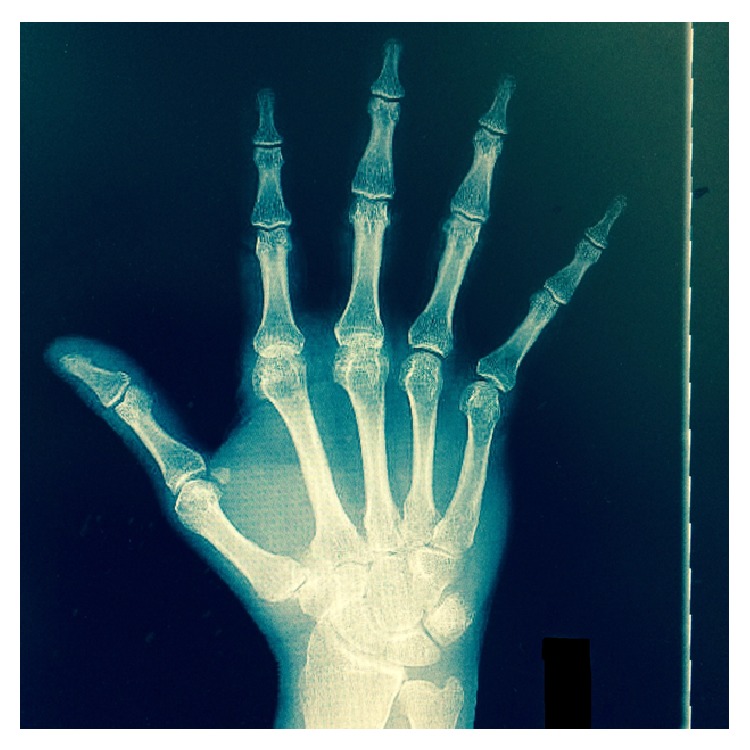
Right windblown hand.

The patient had no history of rheumatoid arthritis, rheumatic fever, or SLE. ESR was 1, uric acid was 6.0, C-reactive protein was 0.0, rheumatoid factor was <10, and he was negative for anti-nuclear antibody suggesting no rheumatoid arthritis or SLE. It was felt to be unlikely seronegative rheumatoid arthritis given his family history and the lack of other symptoms compatible with this disease process. The deformity was not present at birth and he had no other deformities as a child. The pain was isolated to the MP and CMC joints of his hands. Radiographs of his hands were done as seen in Figures 3 and 4. The radiographs confirmed osteoarthritic changes of the left hand and wrist, worsening at the second MCP joint, the third MCP joint, and the scaphotrapezial joint. Spurs were present at the second metacarpal head as well as at the trapezium. The radiographs also confirmed osteoarthritic changes in the right hand with severe joint space narrowing at the scaphotrapezial and first CMC joint, along with minor joint space narrowing in the second and third MCP joints.

## 3. Discussion

Congenital ulnar drift of the fingers was first described by Boix [2] in 1897. In 1932 Lundblom [3] proposed that there was a genetic cause which has been supported by demonstrations of familial occurrence in the literature (Fryns and de Smet [4]; Gavaskar and Chowdary [5]; Grunert et al. [6]). Through these studies it has been widely accepted that windblown hand is inherited in an autosomal dominant manner with variable expression and penetrance. Zancolli and Zancolli Jr. [7] described the condition and developed a scale for the degree of malformations basing the deformity on the malformation of the retaining ligaments or retinaculum of the skin and also on the involvement of other tissues in severe cases. Literature recommends surgery to correct the more severe forms of the deformity before the patient reaches two years of age (Wood and Biondi [8]).

Jaccoud's arthropathy (Jaccoud [9]) was first described in 1869 as a condition that resulted from previous bouts with rheumatic fever and presents with an appearance very similar to that of the windblown hand. Today the condition is still associated with rheumatic fever and also with systemic lupus erythematosus. There have been 2 reported cases of what appears to be idiopathic Jaccoud's arthropathy which presents much like isolated windblown hand but is not noticed until the patient is an adult (Arlet and Pouchot [10]) (Filiz and Yasemin [11]).

In congenital windblown hand the current recommendations are that surgery be performed early in life to avoid later complications (Wood and Biondi [8]). We hypothesize that although our subject had no pain for many years, he may have ultimately become symptomatic secondary to his surgical career and may have benefitted from early correctional surgery. The two brothers that are not surgeons have had no additional pain because of their deviation and seem to represent a subgroup of the affected population that could potentially function without surgery. We conclude that the decision on whether to correct the ulnar deviation of the fingers is complex and must take into consideration the lifestyle of the patient in addition to the severity of the deformity.

Clinically, our patients' hands most resemble idiopathic Jaccoud's arthropathy but are unique in that this has occurred in three siblings. With no clear autosomal dominant pattern present, no memory of this occurring from birth, and no bands felt on extension of the fingers as described by Zancolli, we believe that these three brothers represent a unique presentation of windblown hand.

It is possible that the idiopathic Jaccoud's or adult onset windblown hand is not as rare as literature would imply, but rather since it presents with no pain or loss of function, people affected do not seek treatment or have accepted their physical findings as a normal anatomic variant.
